# The Peripheral Circadian Clock and Exercise: Lessons from Young and Old Mice

**DOI:** 10.5334/jcr.201

**Published:** 2020-12-16

**Authors:** Danielle R. Bruns, Musharraf Yusifova, Nicholas A. Marcello, Carly J. Green, Whitney J. Walker, Emily E. Schmitt

**Affiliations:** 1 College of Health Sciences, University of Wyoming, Laramie, WY, US

**Keywords:** circadian rhythm, exercise, aging

## Abstract

Critical biological processes are under control of the circadian clock. Disruption of this clock, e.g. during aging, results in increased risk for development of chronic disease. Exercise is a protective intervention that elicits changes in both age and circadian pathologies, yet its role in regulating circadian gene expression in peripheral tissues is unknown. We hypothesized that voluntary wheel running would restore disrupted circadian rhythm in aged mice. We analyzed wheel running patterns and expression of circadian regulators in male and female C57Bl/6J mice in adult (~4 months) and old (~18 months) ages. As expected, young female mice ran further than male mice, and old mice ran significantly less than young mice. Older mice of both sexes had a delayed start time in activity which likely points to a disrupted diurnal running pattern and circadian disruption. Voluntary wheel running rescued some circadian dysfunction in older females. This effect was not present in older males, and whether this was due to low wheel running distance or circadian output is not clear and warrants a future study. Overall, we show that voluntary wheel running can rescue some circadian dysfunction in older female but not male mice; and these changes are tissue dependent. While voluntary running was not sufficient to fully rescue age-related changes in circadian rhythm, ongoing studies will determine if forced exercise (e.g. treadmill) and/or chrono-timed exercise can improve age-related cardiovascular, skeletal muscle, and circadian dysfunction.

## Introduction

The mammalian circadian clock governs physiological, endocrine, and metabolic responses coordinated in a 24-hour rhythmic pattern. The suprachiasmatic nucleus (SCN), located in the hypothalamus, is a group of neurons that each contain a molecular clock and together acts as the overall pacemaker to multiple circadian oscillators including those in peripheral tissues where key circadian rhythm genes are expressed [1]. The molecular clock operates on a positive and negative feedback loop system regulated by a number of genes. These genes include CLOCK (Circadian Locomotor Output Cycles Kaput) and BMAL1 (Brain and Muscle ARNT (Aryl hydrocarbon receptor nuclear translocator-like protein-Like1) which are the activator genes that then regulate a number of accessory genes in the molecular clock pathway. The accessory genes include Period1 *(PER1)*, Period2 *(PER2)*, Period3 *(PER3)*, Crytochrome1 *(CRY1)*, and Cryptochromes 2 *(CRY2)* which then feedback to suppress the BMAL/CLOCK heterodimer [2]. *PER2* is arguably the most important of the Period family of genes because *PER2* regulates *PER1* [3]; therefore, *PER2* is considered a core gene of the molecular clock and produces not only circadian rhythm in the SCN but in peripheral tissues as well [4]. The peripheral tissues (i.e. liver, heart, kidney, skeletal muscle, etc.) operate at their own endogenous circadian rhythm but still require the SCN to maintain synchronization [5]. Together, this process dictates key biological functions like neurotransmitter release, heart rate, core body temperature, locomotor activity, feeding schedules, time of rest, and many other diverse functions [6]. Thus, disruption of molecular clock activity is associated with a wide variety of human diseases.

Aging is well-characterized by decreased function of the central clock [7], manifesting most obviously in humans as disrupted sleep-wake cycles [8]. In addition to contributing to poor sleep, attenuated function of the clock contributes to age-related pathologies, as evident in loss of clock function studies in mice, demonstrating cardiac dysfunction [9], sarcopenia [10], and cancer [11], amongst many others. Although peripheral clock activity has also been suggested to decline with age [12], evidence for this hypothesis is lacking, particularly in models of healthy aging.

It is well established that free-wheel running is not only an indicator of circadian activity [5,13], but also a strong predictor of survival from age-related diseases (e.g. cardiovascular disease) [14]. In addition, understanding the molecular mechanisms associated with free wheel running and circadian rhythms is imperative to find interventions which mitigate harmful consequences from circadian misalignment. The peripheral clock can be entrained through exercise; however, the details and extent have yet to be well established. Several lines of evidence suggest that skeletal muscle clock expression can be enhanced through exercise [5,15]; however, many of these findings remain controversial [16]. Thus, the purpose of this study was to compare circadian rhythm regulators (PER2, BMAL, CLOCK) in several peripheral tissues in both young and old mice with and without access to a running wheel. The peripheral tissues analyzed include left ventricle (LV), liver, kidney, and gastrocnemius. We chose these tissues because the peripheral clock differs in each tissue by adapting to its own internal and external stimuli (e.g. feeding cues for the liver) [17] and we aimed to add to the literature to understand how peripheral clocks operate in circadian physiology. We hypothesized that free-wheel running would increase and/or rescue age-related attenuation of core-clock circadian gene expression. Since the disruption of circadian rhythm is linked to a number of human diseases, finding cost-effective solutions (e.g. exercise) to lessening molecular clock dysfunction is imperative to human health.

## Materials and Methods

### Animals

This protocol followed the standards of humane animal care and was approved by the University of Wyoming Institutional Animal Care and Use Committee (#2019-0218ES00339-01). A total of thirty-four C57Bl/6J mice were used in these experiments. The young adult (~4 months) wheel running experimental groups consisted of male (n = 4) and female (n = 6) mice purchased from Jackson Laboratory (Bar Harbor, ME, USA). Young male (n = 4) and female (n = 4) sedentary counterparts were bred in house. Old mice (~18 months) comprised of male (n = 4 experimental and n = 4 sedentary) and female (n = 4 experimental and n = 4 sedentary) wheel running experimental group along with sedentary counterparts were donated from the NIA Aging Rodent Colony (Jackson Laboratory, Bar Harbor, ME, USA). All mice were housed with a consistent 12:12 h light-dark cycle that initiated at 7:00 AM. Food and water were provided ad libitum. The night before sacrifice, wheels were removed from each cage. At the beginning of the light cycle (7 AM), animals were humanely euthanized by an intraperitoneal injection of 100mg/kg of FatalPlus (pentobarbital) and tissues were dissected and flash-frozen for subsequent analysis.

### Voluntary Wheel Running Activity

Mice assigned to the exercise group were singly housed with a running wheel (supplied by Columbus Instruments, Columbus, OH, USA) to be used voluntarily. Each wheel had a magnetic indicator and hall effect sensor that connected to a computer interface and recorded wheel revolutions (converted to kilometers). The wheel had a flat area measuring 2 inches (5.1 cm) × 4 inches (10.2 cm) and had a height of 5.5 inches (14 cm) and an interior diameter of 3.625 inches (9.2 cm). Wheel running data was collected daily, and mice were checked to ensure the wheel was still functioning properly. A five-day introductory period preceded four days of hourly recorded data, ending with five additional days of daily running wheel data for a total of 14 days. Sedentary mice were also individually housed.

### Circadian Gene Expression

RNA was isolated from LV, liver, kidney, and gastrocnemius using standard TRIzol protocols. RNA was reverse transcribed to cDNA via iScript cDNA Synthesis Kit and QuantStudio 5 quantitative real-time PCR (ThermoFisher Scientific) was used to quantify the expression of PER2, BMAL, CLOCK, with β-actin as a housekeeping gene. Data were quantified using the ΔΔ Ct method. Primer sequences can be found in Table 1.

**Table 1:** Primers used in real-time reverse transcriptase-polymerase chain reaction analysis.

Gene	Primers Sequence

PER2	Forward: 5’ – ATGCTCGCCATCCACAAGA – 3’
	Reverse: 5’ – GCGGAATCGAATGGGAGAAT – 3’
BMAL	Forward: 5’ – CCAAGAAAGTATGGACACAGACAAA – 3’
	Reverse: 5’ – GCATTCTTGATCCTTCCTTGGT – 3’
CLOCK	Forward: 5’ – CCAGCACATGATACAGCAAC – 3’
	Reverse: 3’ – GAAGGAAGCTGCTGTTCCTG – 3’
β-Actin	Forward: 5’ – GCAACGAGCGGTTCCG – 3’
	Reverse: 5’ – CCCAAGAAGGAAGGCTGGA – 3’

### Statistical Analyses

Wheel running distance data (kilometers) was analyzed by a one-way ANOVA (four different groups). If the overall model indicated significance, Tukey’s post-hoc analysis was performed to identify statistical differences between groups. Gene expression data were normalized to sedentary animals within sex to first compare the effect of age and then within age and sex to determine the effect of wheel running. Data were compared by Student’s t-test. Significance was set a priori at α < 0.05. Data are presented as means ± SEM. Analyses were performed using JMP Statistics Version 14.1.0 (SAS Corporation, Cary, NC, USA).

## Results

### Wheel Running Distances by Sex and Age

**Figure 1:**
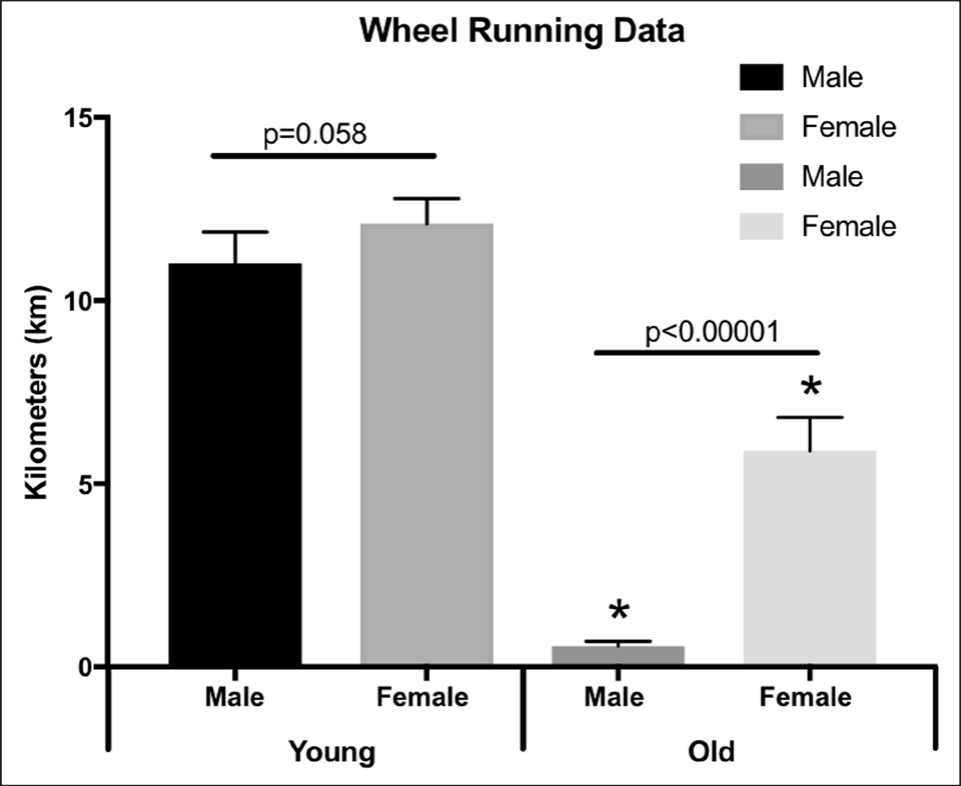
**Running wheel data.** Average wheel running distances over the last 4 days during 14-days of wheel running. Data are shown as means ± SEM (n = 4–6). * p < 0.05.

Running distances were calculated the last four days of the experiment (days 11–14 on the wheel). Overall, though not significant (p = 0.058), young female mice ran more than young male mice averaging 12.10 km ± 0.69 km per night versus 11.02 km ± 0.85 km for the males. Old female mice ran significantly more than old males (p < 0.00001) averaging 5.90 km ± 0.91 compared to 0.57 km ± 0.13 for the males. Old mice ran significantly less compared to young (females p < 0.00001; males p < 0.00001) (Figure 1).

### Hourly Diurnal Circadian Wheel Running Activity

**Figure 2:**
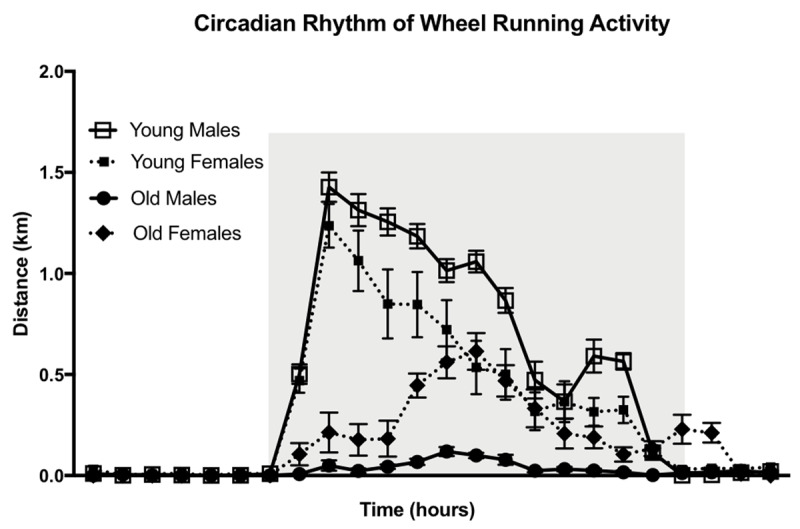
**Circadian rhythm of wheel running activity.** Hourly distance of wheel running in both young and old male and female mice over 24 h compiled during the middle (days 6–9) of the 14-day experiment. Gray shading indicates the dark period when the lights are off. Data are shown as means ± SEM (n = 4–6).

Following wheel acclimation, hourly data was recorded for four days (days six-nine) to determine circadian rhythm of wheel running activity. Young male and female mice began running at the start of the active period as soon as the lights went off, peaking around two hours of darkness. After the initial peak in wheel running activity, young mice had a steady decline in wheel activity that gradually decreased to basal levels when the lights turned back on. Old male and female mice demonstrated a delay in wheel activity, peaking at six (males) and seven (females) hours into the dark phase. In addition, old female mice recorded running wheel revolutions two hours into the light phase, demonstrating a delayed return to basal levels compared to younger animals (Figure 2).

### Circadian Expression of Clock-Controlled Genes in Peripheral Tissues

**Figure 3:**
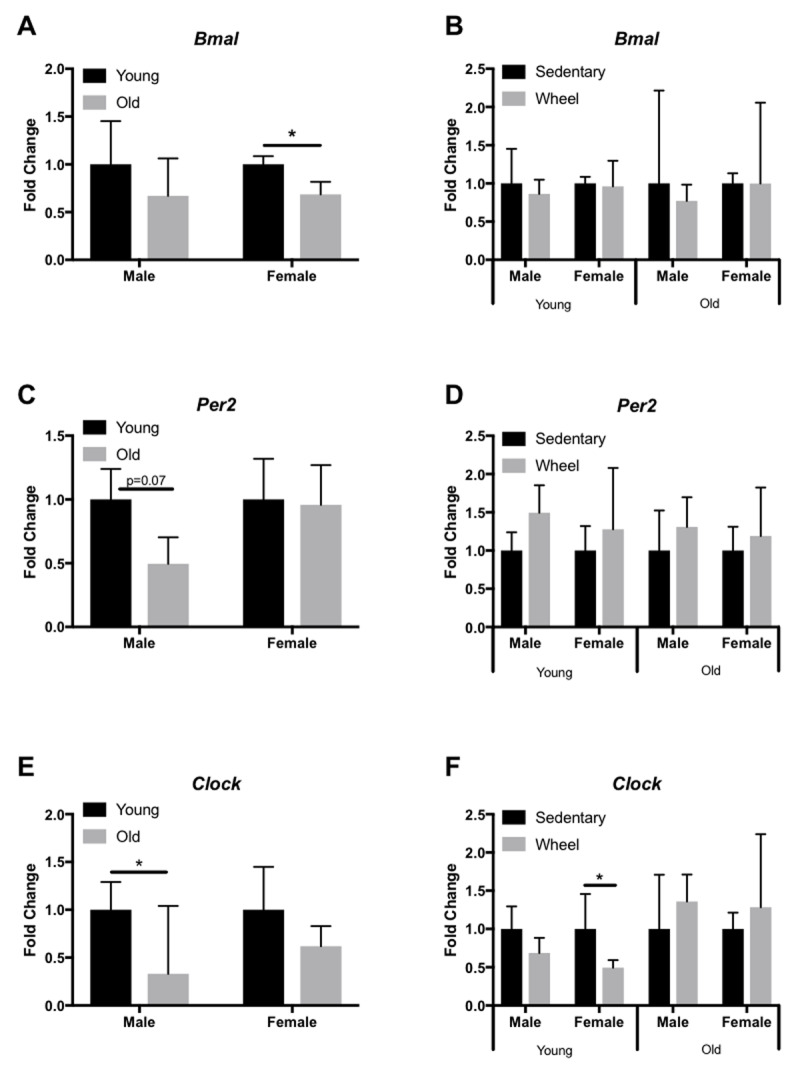
**Circadian expression of clock-controlled genes in the mouse liver.** Gene expression was quantified by qRT-PCR and normalized to β-Actin. Samples were collected at 7am. Data are represented as the effect of age (A,C,E) and the effect of wheel running (B,D,F). Data were assessed by Student’s t-test. Data are expressed as means ± SEM. * p < 0.05. (n = 3–4).

**Figure 4:**
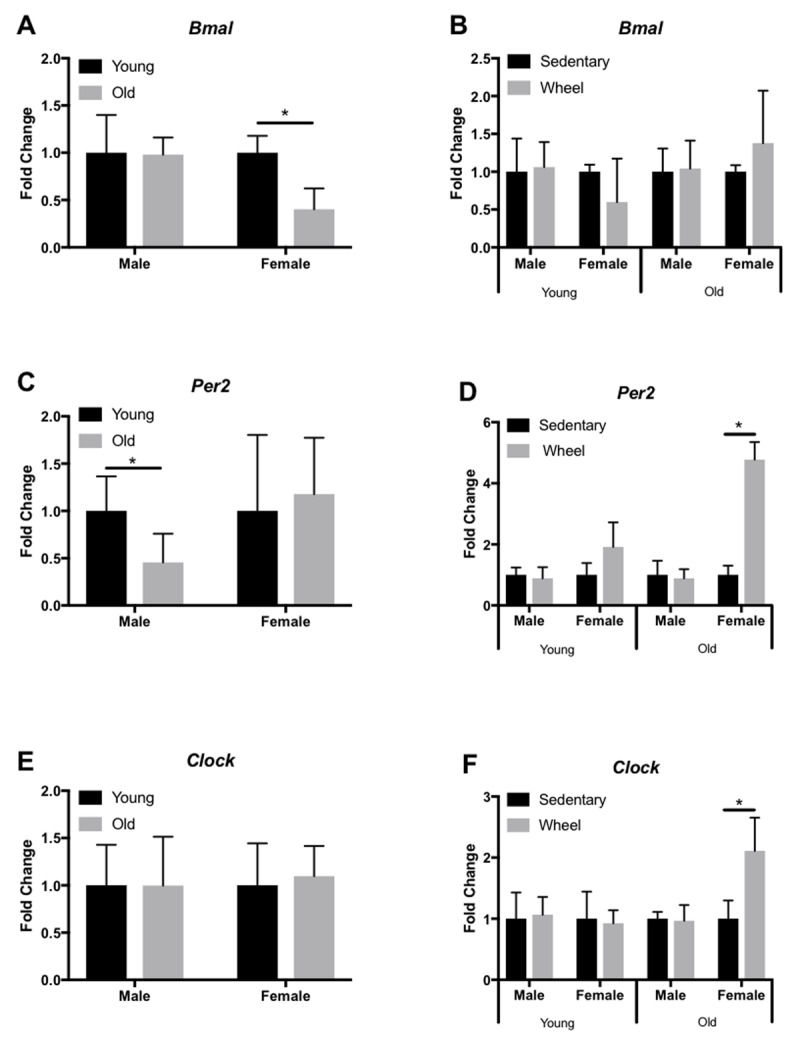
**Circadian expression of clock-controlled genes in the mouse LV.** Gene expression was quantified by qRT-PCR and normalized to β-Actin. Samples were collected at 7am. Data are represented as the effect of age (A,C,E) and the effect of wheel running (B,D,F). Data were assessed by Student’s t-test. Data are expressed as means ± SEM. * p < 0.05. (n = 3–4).

**Figure 5:**
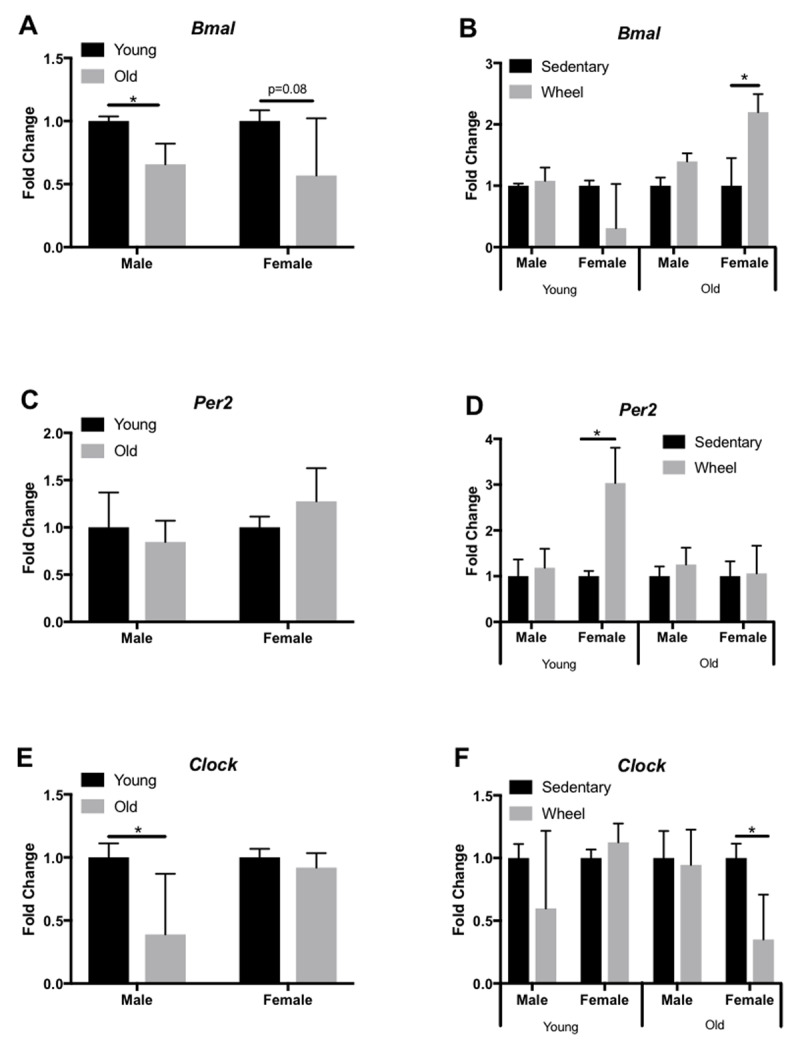
**Circadian expression of clock-controlled genes in the mouse kidney.** Gene expression was quantified by qRT-PCR and normalized to β-Actin. Samples were collected at 7am. Data are represented as the effect of age (A,C,E) and the effect of wheel running (B,D,F). Data were assessed by Student’s t-test. Data are expressed as means ± SEM. * p < 0.05. (n = 3–4).

**Figure 6:**
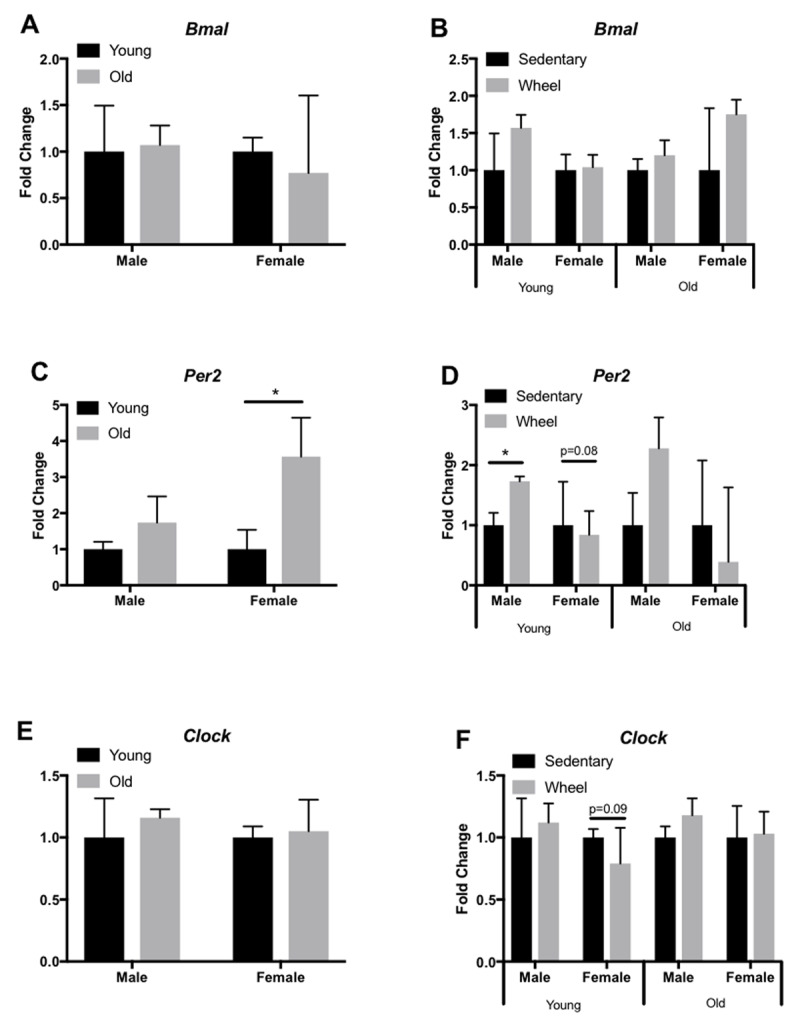
**Circadian expression of clock-controlled genes in the mouse gastrocnemius.** Gene expression was quantified by qRT-PCR and normalized to β-Actin. Samples were collected at 7am. Data are represented as the effect of age (A,C,E) and the effect of wheel running (B,D,F). Data were assessed by Student’s t-test. Data are expressed as means ± SEM. * p < 0.05. (n = 3–4).

We analyzed expression profiles of several circadian clock genes (*Bmal, Per2, Clock*) in the liver, LV, kidney, and gastrocnemius via qRT-PCR. In the liver, old female mice only showed a significant decrease in *Bmal* expression (Figure 3A), yet old male mice had a significant decrease in *Clock* expression (Figure 3E). However, young female mice had a significant decrease in *Clock* expression in the liver when given access to a running wheel (Figure 3F). In the LV, old female and male mice show a significant decrease in *Bmal* (Figure 4A) and *Per2* (Figure 4C) expression, respectively; however, *Clock* expression did not change with age (Figure 4E). When analyzed by group (sedentary vs wheel running), there was no change in *Bmal* expression in either sex (Figure 4B), yet there was a significant increase in *Per2* (Figure 4D) and *Clock* (Figure 4F) in old females that ran on a wheel. In the kidney, old male mice had a significant decrease in *Bmal* (Figure 5A) and *Clock* (Figure 5E) expression when compared to young males. When given access to a running wheel, old female mice had significantly higher expressions of *Bmal* (Figure 5B), yet a significantly lower expression of *Clock* (Figure 5F). Young females had a significantly greater expression of *Per2* in the kidney when compared to sedentary counterparts of the same sex (Figure 5D). Finally, in the gastrocnemius, young male mice with access to a running wheel had a higher expression of *Per2* (Figure 6D) with no change in *Bmal* (Figure 6B) or *Clock* (Figure 6F) proteins when compared to sedentary counterparts. In addition, young female mice that exercised also showed no change in *Bmal*, yet a trend (p = 0.08) towards a decrease in *Per2* expression (Figure 6D) and a trend (p = 0.09) towards a decrease in *Clock* expression (Figure 6F) when compared to animals that did not exercise. The only significant change noted in old mice in the gastrocnemius was an increase in *Per2* expression in females (Figure 6C). Gene expression results in all tissues analyzed by age, sex, and activity status can also be found summarized in Table 2 and Table 3.

**Table 2:**
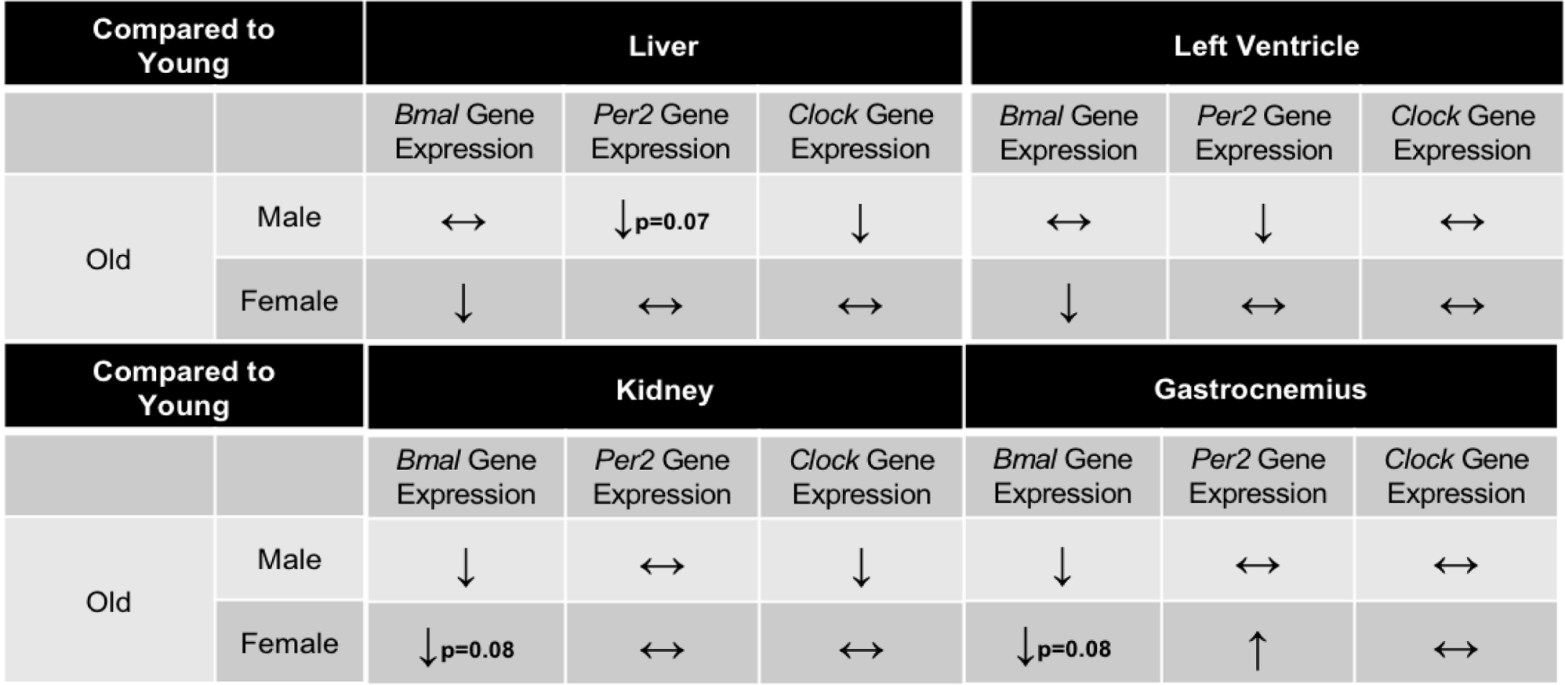
**Summary table of circadian gene expression in old mice.** Summary of changes in *Bmal, Per2*, and *Clock* expression in Liver, Left Ventricle, Kidney, and Gastrocnemius mouse tissues. Data represents old mice compared to young mice. ↔ indicates no change in gene expression, ↑ indicates a significant increase in gene expression, ↓ indicates a significant increase in gene expression.

**Table 3:**
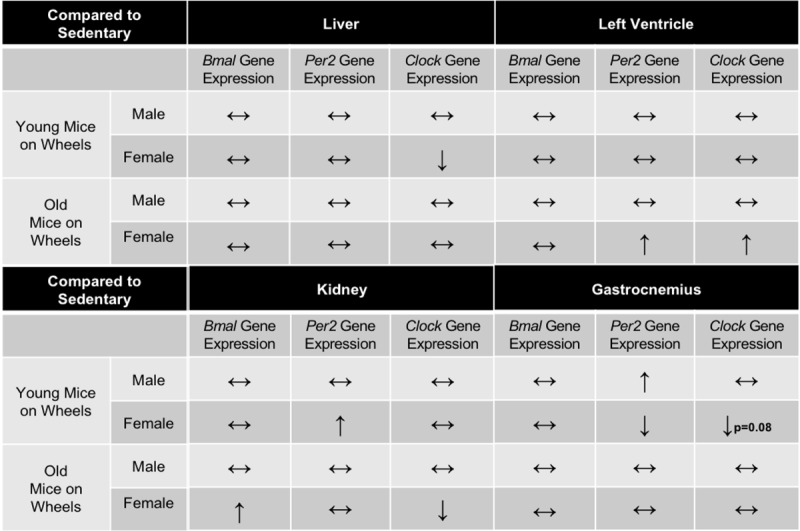
**Summary table of circadian gene expression in active mice.** Summary of changes in *Bmal, Per2*, and *Clock* expression in Liver, Left Ventricle, Kidney, and Gastrocnemius mouse tissues. Data represents young mice with access to wheels compared to sedentary age-matched controls. ↔ indicates no change in gene expression, ↑ indicates a significant increase in gene expression, ↓ indicates a significant increase in gene expression.

## Discussion

Circadian rhythms govern critical biological processes, and this control diminishes with age. Voluntary exercise represents a means to reset these age-associated declines in clock activity through action on peripheral clock proteins to control physiological circadian rhythms [18]. Here, we tested the hypothesis that aging is characterized by declines in peripheral clock expression, and that voluntary wheel running would attenuate these declines. We analyzed circadian clock expression profiles in the liver, LV, kidney, and gastrocnemius. We chose these peripheral tissues because of differing internal and external cues regulating each tissue independently. Our results indicate that aging does affect circadian related genes, but in a sex and tissue- specific manner (Table 2, 3, Figures 3, 4, 5, 6). Overall, aged males demonstrate more circadian dysfunction than aged females, and these differences vary throughout peripheral tissues in the mouse. We show that wheel running can rescue some circadian dysfunction in older female, but not male mice, if given access to a running wheel; yet these changes are also tissue dependent. Ongoing and future experiments will aim to elucidate if forced exercise in old mice can rescue circadian misalignment in aged mice. Our group continues to aim to use exercise as medicine to reset peripheral molecular clocks that suffer from circadian misalignment, whether that is due to advanced age, chronic jet lag, or shift work.

Mammalian peripheral circadian clocks are controlled by the SCN but have their own tissue level autonomy and operate independently of each other. Studies generally focus on one peripheral tissue analysis but our group wanted to test on a more global scale, the effect of exercise and age on several peripheral circadian tissues. Of the peripheral tissues, most work to date has focused on skeletal muscle molecular clocks and exercise. Though limited, mice who voluntarily exercised had significant shifts in PER2::LUC bioluminescence rhythms suggesting that the circadian clock in the skeletal muscle responds to exercise [5]. We also know that skeletal muscle function declines as we age, which begs the question if exercise can truly reset the clock even in advancing age. Our group found in the gastrocnemius no change in *Bmal* expression regardless of age, sex, or running wheel access. However, we did find changes in *Per2* and *Clock* expression in female mice, with the most significance in an increase in *Per2* expression in old female mice (Figure 6C). These results suggest that, at least in old female mice, there are changes in the molecular clock as a result of aging. The heart is under circadian control and evidence suggests that circadian rhythm alterations that regulate physiological functions may contribute to cardiovascular disease risk [19]. Though age-related declines in cardiac circadian rhythm have been reported with respect to heart rate [20] and blood pressure [21], the expression of core clock genes has not been reported, nor has the impact of exercise on restoring these age-associated disrupted rhythms. Here, we analyzed *Per2, Clock, and Bmal* expression in the LV and found disrupted expression with age, but in a sex-specific manner. Voluntary wheel running appeared to mildly rescue this phenotype, but only in female mice. Regular exercise is among the most potent cardioprotective interventions yet identified [22] and cardiorespiratory fitness strongly predicts survival [23]. However, despite the strong links between exercise and cardiac health, the molecular mechanisms by which exercise helps the heart, specifically the aged heart, are not fully elucidated. We suggest that future investigations aim to understand cardiac circadian rhythm, and the mechanisms by which exercise may help restore clock function, particularly in the heart.

Alternations in kidney circadian rhythms can lead to a multitude of abnormalities in renal function [24,25,26]. Regulation of renal blood flow and fluid balance, as well as removing waste and metabolites from the blood are all important functions of the kidney that decline with advanced age. Exercise as a means to reset the molecular clock has been proposed by our group [26]. We previously found that *Per2* expression does not change across the lifespan in males but significantly declines after middle age in females [26]. The current study assessed if exercise could add to changes in molecular clock expression and we did find that young females who voluntarily ran had higher expression of *Per2* compared to sedentary counterparts. In older females, mice who exercised had significantly higher levels of *Bmal* expression, yet down regulated levels of *Clock* expression. Others have found an inverse relationship where *Bmal* expression is down regulated with *Clock* expression upregulated in the kidneys [27], but in male mice who did not exercise. These differences point to the understanding that males and females display different circadian rhythms in peripheral tissues and these rhythms can be impacted by exercise as evident by changes in gene expression.

The circadian clock also plays a crucial role in activation of metabolic pathways, and metabolism relies on clock coordination of hormone regulation and enzyme activation to carry out important physiological functions like energy homeostasis. Therefore, the liver provides a dynamic insight into the coordination of these synchronized metabolic processes. In the current study, analysis of circadian clock expression in the liver demonstrated significant changes in gene expression. For example, old female mice had a decrease in *Bmal* expression when compared to young counterparts, and young female mice who exercised had a significant decrease in *Clock* compared to age-matched controls. Interestingly, male mice had a trend towards a significant (p = 0.07) decline in *Per2* expression and a significant decline in *Clock* expression. It is not surprising that the differences in age and activity levels between animals resulted in differences in core clock gene expression. As organisms age, molecular clock disruption can affect well-synchronized mechanisms in metabolism, resulting in disrupted feeding and fasting cycles in mice [28].

We chose voluntary wheel-running for these experiments, based on previous work demonstrating robust changes in the skeletal muscle molecular clock from exercise [5,15,29]. Exercise not only elicits specific changes in the skeletal muscle molecular clock, but can also cause activation or repression of signaling pathways that regulate protein synthesis or degradation [30]. However, different modalities of exercise warrant further discussion. Our data demonstrate a significant effect of both sex and age on voluntary wheel running, with young males running slightly more than young females on days six-nine of the experiment (Figure 2), but young females out-running young males by the end of the 14-day wheel running experiment (Figure 3). However, aged animals running significantly less than young. No significant differences in clock related proteins were detected in old male mice regardless of wheel exposure in any peripheral tissues tested because old male mice do not use the wheel enough to elicit any positive changes, averaging only a short distance of 0.57 km/day. Therefore, it is possible that exercise did not impact expression of peripheral circadian rhythm genes in the aged male animals due to low exercise volume, intensity, or duration. It is therefore likely that a forced exercise protocol, such as treadmill running or swimming, would elicit changes in peripheral circadian gene expression, especially in old male mice. The specific timing of exercise may also be important (e.g. forced running or swimming at specific times of day), as demonstrated in transcriptome and metabolome data in skeletal muscle from mice exercised early in the rest phase or early in the active phase [29].

### Limitations

These experiments were conducted at 7,220 feet (2.2 kilometers) above sea level. The literature to date (i.e. voluntary running wheel distances) has been published from labs located at lower elevations. At sea level, C57BL/6J females average approximately 8.4 km/day, with males running 6.8 km/day [31]. In our hands, adult females averaged 12.1 ± 0.69 km/day and adult males 11.0 ± 0.85 km/day. At 40 weeks of age, old females at sea level have been reported to run approximately 4.5 km/day, with aged males running 3.5 km/day [32]. Our mice are considerably older than this report at ~78 weeks, and averaged 5.90 ± 0.91 km/day for females and 0.57 ± 0.13 km/day for males. Thus, it is likely that altitude impacts running wheel distances, particularly in the aged mice. Ongoing work from our group aims to elucidate the impact of high altitude and hypoxia on wheel-running and circadian gene expression in different mouse strains across the lifespan and in both sexes.

### Conclusions

Voluntary wheel running for two weeks can rescue age related decline in circadian rhythm in female but not male mice in a tissue-specific manner. These results indicate that peripheral clocks can be reset through exercise, but sex specific and age differences must be considered. Males and females age at different rates; therefore, circadian gene expression in peripheral tissues will likely vary by age as well. Understanding how the SCN governs peripheral molecular clocks, how the peripheral clocks exhibit internal tissue-level diurnal activity, and how exercise can reset the molecular clock are imperative to move the field of circadian chronobiology forward.
